# Report of a rare case: occult hemothorax due to blunt trauma without obvious injury to other organs

**DOI:** 10.1186/1749-8090-8-205

**Published:** 2013-11-01

**Authors:** Fumihiro Ogawa, Masahito Naito, Akira Iyoda, Yukitoshi Satoh

**Affiliations:** 1 Department of Thoracic Surgery, Kitasato University School of Medicine, 1-15-1 Kitasato, Minami-ku, Sagamihara, Kanagawa 252-0374, Japan; 2 Department of Thoracic Surgery, Toho University School of Medicine, Tokyo, Japan

**Keywords:** Hemothorax, Trauma, Inferior phrenic artery, Diaphragm rupture

## Abstract

Traumatic hemothorax commonly occurs accompanied by organ damage, such as rib fractures, lung injury and diaphragm rupture. Our reported patient was a 61-year-old man who fell down from a stepladder about 1 meter in height, resulting in a heavy blow to the left abdomen. He consulted a clinic because of left chest pain the next day and was transported to the emergency center of our hospital on diagnosis of hemothorax with hemorrhagic shock.

On computed tomography scanning with contrast medium, left hemothorax without rib fracture, diaphragm rupture or obvious organ injury was evident. We found only bleeding to the thoracic space from a branch of the left inferior phrenic artery without involvement of the abdomen. The patient underwent percutaneous angiography and embolization for hemostasis, and subsequently thoracotomy in order to check the active bleeding and remove the hematoma to improve respiratory. As thoracotomy findings, we found damage of a branch of the left inferior phrenic artery to the thoracic space without diaphragm rupture, and sutured the lesion. Such active intervention followed by surgical procedures was effective and should be considered for rare occurrences like the present case. We must consider not only traumatic diaphragm rupture, but also vascular damage by pressure trauma as etiological factors for hemothorax.

## Introduction

Traumatic hemothorax commonly occurs immediately after trauma, accompanied by organ damage, such as rib fracture, lung injury and diaphragm rupture [1]. Importantly, hemothorax with diaphragm rupture is reported to be generally not fatal, but the mortality rate reaches 18-50% when complicated with great vessel injury and involvement of major organs. In such cases it requires immediate diagnosis and medical intervention [1].

We here report a rare case in which occult hemothorax occurred without organ injury.

## Case presentation

A 61-year-old man fell down from a stepladder about 1meter in height and suffered a heavy blow to the left abdomen during performance of carpenter’s work. Next day, he went to a clinic because of continued left chest pain. In a chest X-ray, fluid effusion in his left thoracic space without pneumothorax was noted and he was transported to the emergency center of our hospital based on diagnosis of hemorrhagic shock with hemothorax.

**Figure 1:**
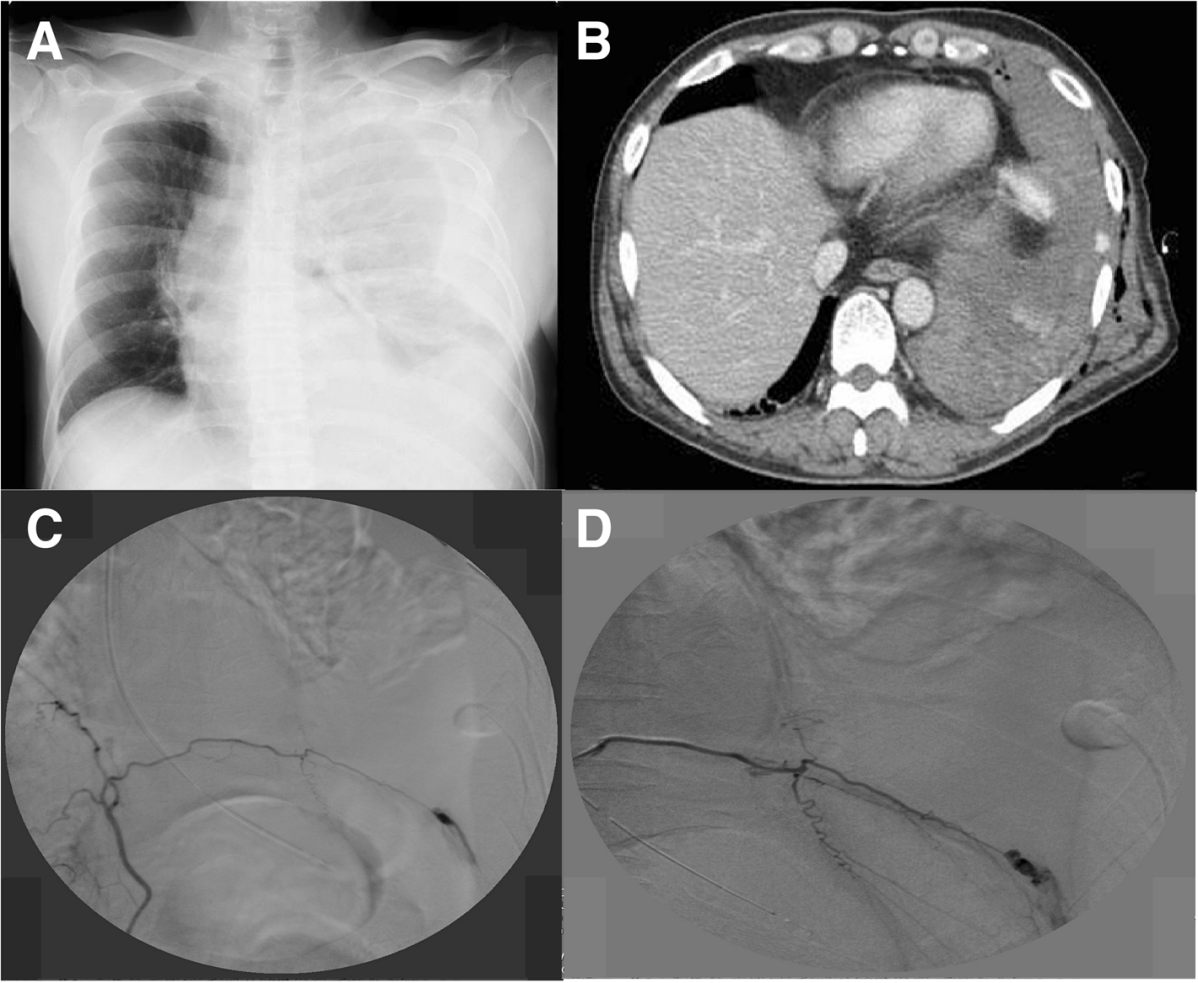
**Preoperative images. (A)** Chest X-ray film showing left tension hemothorax; **(B)** Enhanced chest computed tomography (CT) shows left hemothorax and bleeding into the thoracic space from a branch of the left inferior phrenic artery without involvement of the abdomen in the delay phase; **(C)** Note the leak of contrast medium into the thoracic space on angiography of the selected left phrenic artery; **(D)** Bleeding was markedly reduced after coil embolization.

**Figure 2:**
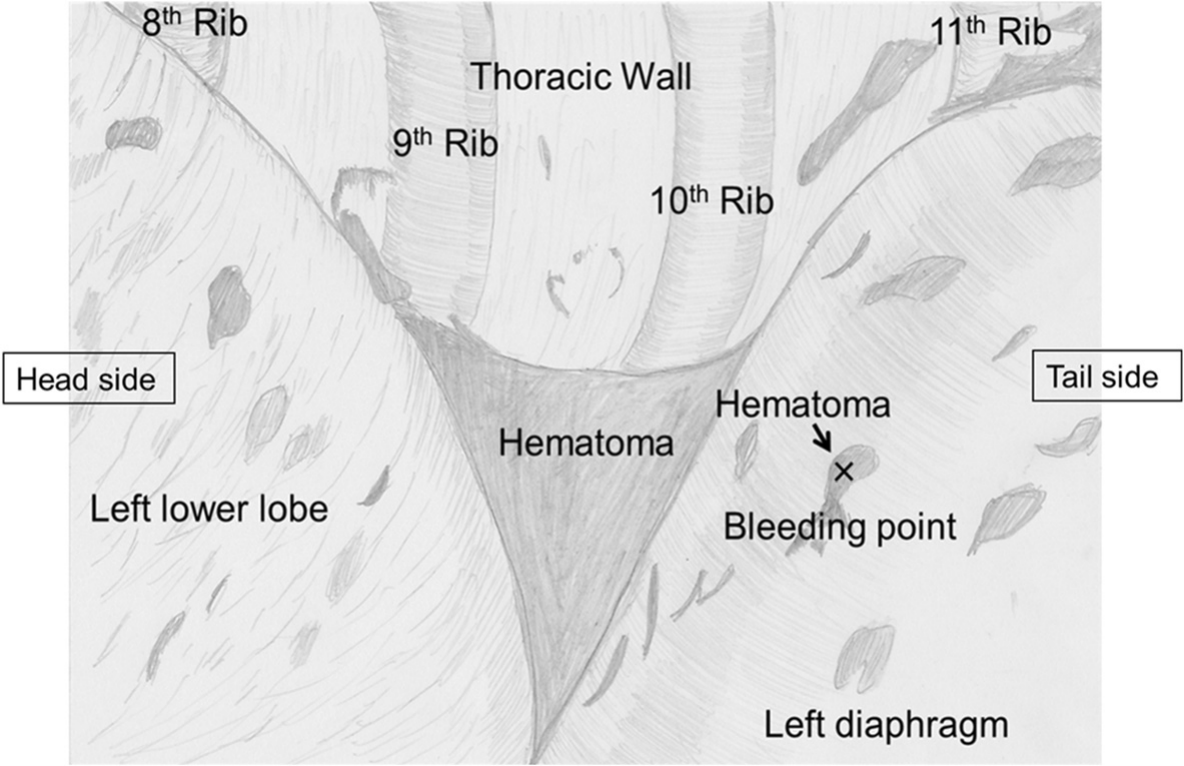
**Intra-operative schematic illustration of the left thoracic cavity.** Note the blood vessel considered to be a branch of the inferior phrenic artery of the diaphragm surface without obvious lung damage, rib fracture or diaphragm rupture.

In chest x-rays (Figure 1A), the left tension hemothorax was apparent when he arrived at our institution. He underwent left chest drainage and about 1,500 ml blood was drained. After releasing tension hemothorax, we performed enhanced chest computed tomography (CT) scanning and found only bleeding into the thoracic space from a branch of the left inferior phrenic artery without involvement of the abdomen in the delay phase (Figure 1B). Additionally, obvious lung injury, rib fracture or diaphragm rupture were not found. Therefore, we performed interventional radiology (IVR) for continuous bleeding control before thoracotomy. Since we confirmed bleeding from the left phrenic artery by IVR (Figure 1C), coil embolization was performed and the bleeding gradually reduced (Figure 1D). However, emergency thoracotomy was planned due to further bleeding of about 800 ml, serious anemia (hemoglobin decrease from 11.3 g/dl to 7.1 g/dl), hypotension through hemorrhagic shock and respiratory disorder due to a huge hematoma in the thoracic space. As the finding of left thoracotomy (Figure 2), we located the blood vessel thought to be a branch of the inferior phrenic artery of the diaphragm surface without obvious lung damage, rib fracture or diaphragm rupture, in line with the preoperative images. When oozing was confirmed from the site, it was sutured with three 1–0 silk stitches, and on confirmation of no further bleeding, thoracotomy was completed with MAP 6 U infusion to improve serious anemia.

The postoperative course was uneventful and he could be discharged from hospital without complications on the 6^th^ day after thoracotomy.

## Discussion

This is very rare case of hemothorax without multiple organ damage after brunt trauma, unlike any prior example that we could find in the literature. It was previously reported that hemothorax is produced when blunt trauma causes dome top lesions to the diaphragm from the abdominal side in cases of diaphragm rupture [1,2]. Lateral collisions, which are three times more common, cause ipsilateral tears secondary to thoracic distortion and shearing [2]. In this case, there was no diaphragm rupture, but it was thought that intraphrenic artery damage resulted because a remarkable pressure gradient was applied to the diaphragm. Traumatic diaphragm injury accounts for 0.8 ~ 3.3% of blunt trauma cases [2,3], and when restricted to traffic accidents, the frequency of the diaphragm rupture is relatively rare at 1-5% [2,4]. In fact, the reason for traumatic diaphragm injury was previously reported to be traffic accidents in about 70% of cases, and, interestingly, left sided rupture is three times more common than on the right [3,5]. Cadaveric studies have demonstrated that the pressure required to rupture the left hemidiaphragm is consistently lower than that on the right [6], due to the relative weakness on the left from the lumbocostal trigone to the point of embryological fusion.

Since patients with traumatic lesions and hemorrhagic pleural effusion usually have multiple bleeding sources, contrast-enhanced CT is generally considered necessary to identify the bleeding points, document their anatomic relationships, and detect extravasation of contrast agent or pseudoaneurysms [2,7,8].

Transcatheter arterial embolization is commonly considered the most reliable and feasible therapeutic alternative to thoracotomy for control of intrathoracic arterial hemorrhage [9,10].

Carrillo et al. stated that the morbidity associated with thoracotomy, coupled with the frustratingly low likelihood of finding the source of hemorrhage in some patients, makes selective angiography and transcatheter embolization a less invasive, more accurate, and reliable method for treatment [9]. Another problem is disorder of the clotting system, which is generally induced by massive hemorrhage and may develop into consumption coagulopathy in patients with multiple bleeding sources. This potentially lethal disorder is difficult to treat and results in uncontrollable diffuse bleeding.

## Conclusions

In conclusion, enhanced CT scan and IVR are effective procedures for identifying the origin of bleeding into the thoracic space without diaphragm rupture or obvious organ injury. In addition, conclude that active intervention, such as surgical procedures, may be necessary for rare occurrences like the present case. We must consider not only traumatic diaphragm rupture, but also vascular damage by pressure trauma as etiological factors for hemothorax.

## Consent

Written informed consent was obtained from patient for publication of this case report and any accompanying images. A copy of the written consent is available for review by the Editor-in-Chief of this journal.

## Competing interests

The authors declare that they have no competing interests.

## Authors’ contributions

FO carried out the manuscript and collected references. YS coordinated all authors. FO, MN and AI underwent this operation and helped for clinical support with them. AI and YS helped to draft the manuscript. All authors read and approved the final manuscript.
